# Self-Administered Domiciliary tDCS Treatment for Tinnitus: A Double-Blind Sham-Controlled Study

**DOI:** 10.1371/journal.pone.0154286

**Published:** 2016-04-28

**Authors:** Petteri Hyvärinen, Antti Mäkitie, Antti A. Aarnisalo

**Affiliations:** 1 Department of Otorhinolaryngology—Head and Neck Surgery, University of Helsinki and Helsinki University Hospital, Biomedicum Helsinki 1, P.O. Box 220, FI-00029 HUS, Helsinki, Finland; 2 Department of Neuroscience and Biomedical Engineering, Aalto University School of Science, P.O. Box 12200, FI-00076 AALTO, Espoo, Finland; University Medical Center Goettingen, GERMANY

## Abstract

Transcranial direct current stimulation (tDCS) has shown potential for providing tinnitus relief, although positive effects have usually been observed only during a short time period after treatment. In recent studies the focus has turned from one-session experiments towards multi-session treatment studies investigating long-term outcomes with double-blinded and sham-controlled study designs. Traditionally, tDCS has been administered in a clinical setting by a healthcare professional but in studies involving multiple treatment sessions, often a trade-off has to be made between sample size and the amount of labor needed to run the trial. Also, as the number of required visits to the clinic increases, the dropout rate is likely to rise proportionally.The aim of the current study was to find out if tDCS treatment for tinnitus could be patient-administered in a domiciliary setting and whether the results would be comparable to those from in-hospital treatment studies. Forty-three patients with chronic (> 6 months) tinnitus were involved in the study, and data on 35 out of these patients were included in final analysis. Patients received 20 minutes of left temporal area anodal (LTA) or bifrontal tDCS stimulation (2 mA) or sham stimulation (0.3 mA) for ten consecutive days. An overall reduction in the main outcome measure, Tinnitus Handicap Inventory (THI), was found (mean change *−*5.0 points, *p < 0*.*05*), but there was no significant difference between active and sham treatment outcomes. Patients found the tDCS treatment easy to administer and they all tolerated it well. In conclusion, self-administered domiciliary tDCS treatment for tinnitus was found safe and feasible and gave outcome results similar to recent randomized controlled long-term treatment trials. The results suggest better overall treatment response—as measured by THI—with domiciliary treatment than with in-hospital treatment, but this advantage is not related to the tDCS variant. The study protocol demonstrated in the current study is not restricted to tinnitus only.

## Introduction

Tinnitus is an auditory disorder defined by a subjective phantom sound sensation and it affects 10–15% of the adult population [1,2]. Often tinnitus develops as a result of trauma to the auditory periphery, such as noise-induced hearing loss or an auditory nerve lesion [1]. Clinically positive symptoms of hearing loss are not a necessary precondition for tinnitus, but recent studies have suggested that some forms of hearing loss underlying tinnitus can go unnoticed by the current standard diagnostic tools [3,4]. The damage to the early stages of the auditory pathway is thought to set in motion a series of plastic changes along the whole auditory chain, eventually leading to a tinnitus sensation in some subjects [5,6]. Tinnitus-related structural changes as well as alterations in spontaneous and evoked activity patterns have been detected not only throughout the auditory system, but also in brain areas responsible for non-auditory processing [7–16]. Therefore, tinnitus is currently seen as a complex disorder involving multiple overlapping brain networks and it is widely accepted that there is likely no well-defined neural substrate for tinnitus to be found.

Due to the association between tinnitus and the structural and functional changes seen in the central nervous system, the affected brain areas have often been chosen as targets for various neurostimulation and neuromodulation interventions. The aim is usually to modulate either the tinnitus percept or its affective components by disrupting the underlying pathological neural activity. Immediate positive responses in tinnitus patients have been achieved with tDCS, targeting either the dorsolateral prefrontal cortex (DLPFC) or the auditory cortex [17], but the observed effects have not translated into long-term improvements. Explorative analyses have suggested that women [18], subjects with a lower degree of hearing loss [19], and subjects with enhanced auditory gamma-activity [20] might respond better to tDCS.

TDCS is normally administered by a healthcare professional in a clinical setting. This ensures minimal inter-individual variability in the procedure and most importantly correct administering of the treatment [21]. The downside of in-hospital treatment is that the subject has to visit the hospital separately for each session in case they are not already in the hospital for other reasons [22]. The increased effort needed to participate in a study might make recruitment from certain patient groups more difficult, or increase the drop-out rate in long-term studies [23]. When moving from early open-label pilot studies to sham-controlled trials, it is beneficial to have the option of at-home treatment. The only study involving self-administered at-home tDCS stimulation, that the authors are aware of, investigated pain treatment and reported positive outcomes and no adverse effect [24]. There was however a relatively high drop-out rate in that study, with three subjects out of 17 discontinuing treatment because of experienced difficulty in application of the stimulation. Also Charvet et al. (2015) [22] identify the ease of electrode preparation and positioning as essential factors in reproducible remotely-supervised tDCS treatment.

The current study aims to provide a safe and feasible protocol for patient-administered at-home tDCS treatment for tinnitus, and to investigate if this will be achieved with treatment outcomes comparable to in-hospital treatment. Special attention was given to user experience aspects of the treatment, since a major factor in successful and reproducible tDCS treatment is in the correct preparation of the stimulation.

## Materials and Methods

### Subjects

Forty-three patients (20 female; average age 51.0 years, SD 15.4 years) were recruited to the study (Table 1, Supporting Data). The study ran from December 2013 to September 2015. All participants were patients of the Hearing Center at the Department of Otorhinolaryngology–Head and Neck Surgery, Helsinki University Hospital, Helsinki, Finland and had been referred to the Hearing Center by general practicioners because of bothersome tinnitus. None of the patients had any previous experience with tDCS. Patients underwent standard audiological evaluation by an ENT doctor and gave written informed consent before enrolling in the study. Only patients with chronic tinnitus—i.e. tinnitus that had continued for over six months—were included in the study. Contraindications for participation in the study were epilepsy, migraine, and implants in the head and neck area. Patients with high levels of tinnitus distress were assumed to benefit more from the treatment, so in those cases where the patient had completed the THI questionnaire earlier, we aimed at recruiting patients with THI scores of 18 or more. If there was no earlier THI score, the recruitment was based on the willingness of the patient. All patients scoring less than 18 points on the baseline THI questionnaire—indicating slight tinnitus distress [25]—were assigned to a control group, and excluded from the statistical analyses. Their results are however shown in order to give a point of reference for comparison and to indicate possible THI-score-related trends. The study was approved by the Research Ethics Board at the Helsinki University Hospital and conducted in line with the Declaration of Helsinki.

**Table 1:** Patient characteristics of the participating subjects. T is short for tinnitus. Tinnitus side is marked with the following logic: L: left, R: right, R = L: bilateral with no lateralization, R>L: bilateral lateralizing more to the right side, L>R: bilateral lateralizing more to the left side. Italicized rows represent patients who were excluded from final analysis (see *Results* section).

***Bifrontal***									
**ID**	**age**	**sex**	**handed-ness**	**T side**	**T type**	**T cause/initial event**	**PTA L/R**	**THI**	**ΔTHI**
B1	74	F	R	NA	musical + hissing	Not known	66/59	38	−10
B7	56	F	R	L	mid-freq whooshing	loss of balance	NA	36	−16
B11	66	F	R	NA	hissing + ringing	Not known	12/10	24	+4
*B15*	*35*	*M*	*R*	*R = L*	*high tonal + hissing*	*Not known*	*NA*	*62*	*NA*
*B17*	*53*	*F*	*R*	*R*	*humming*	*sudden deafness*	*15/76*	*66*	*−52*
B24	73	F	R	L	buzzing	Not known	31/26	36	−26
B25	47	M	both	L>R	high tonal	work-related sound exposure	6/2	42	+4
B26	59	M	R	L>R	hissing, whistling	Not known	19/17	36	−22
B29	54	F	R	L	high tonal	Not known	19/5	36	−4
B34	53	F	R	L = R	crickets + low pulsating	sound exposure	23/26	78	+10
B36	37	F	R	L>R	hissing + low whooshing	Not known	8/12	36	+6
*B43*	*68*	*M*	*R*	*L>R*	*hissing*	*Not known*	*28/43*	*8*	*+16*
***LTA***									
**ID**	**age**	**sex**	**handed-ness**	**T side**	**T type**	**T cause/initial event**	**PTA L/R**	**THI**	**ΔTHI**
L2	55	M	NA	L>R	high tonal	Not known	48/18	28	−4
*L4*	*54*	*M*	*R*		*high tonal*	*Not known*	*NA*	*44*	*NA*
L8	67	F	R	L	low whooshing + high tonal	Not known	28/24	30	+10
L10	25	M	R	R>L	multiple high tones	sound exposure	4/7	62	−28
L13	18	F	R	L = R	multiple high tones	sound exposure	−2/1	48	+6
L20	29	M	R	L = R	high tonal	Not known	5/−1	22	−10
*L27*	*64*	*M*	*R*	*L*	*hissing*	*Not known*	*NA*	*58*	*NA*
L28	38	M	R	L = R	high tonal	work-related sound exposure	−1/2	20	+6
L31	46	M	R	L = R	whistling	Not known	24/21	70	−6
L32	46	M	R	R>L	high tonal	Not known	6/5	40	+2
*L33*	*49*	*F*	*L*	*L = R*	*high tonal*	*Not known*	*10/4*	*26*	*NA*
*L35*	*36*	*F*	*R*		*hissing*, *whistling*, *ringing*	*sudden deafness*	*NA*	*62*	*NA*
L38	58	M	both	L = R	high tonal	Not known	54/58	18	−8
*L39*	*51*	*M*	*R*	*L = R*	*hissing*, *high tonal*	*Not known*	*14/9*	*88*	*−24*
L42	20	F	R	R	hissing	Not known	4/19	34	−12
***Sham***									
**ID**	**age**	**sex**	**handed-ness**	**T side**	**T type**	**T cause/initial event**	**PTA L/R**	**THI**	**ΔTHI**
S3	57	M	NA	L>R	hissing	sound exposure	24/35	52	+4
S5	50	M	R	L>R	high tonal	Not known	13/20	44	+4
S12	57	M	R	NA	multiple high tones + high buzzing	Not known	3/6	28	−2
S14	71	M	R	L>R	humming	Not known	54/54	58	−14
S18	69	M	R	L = R	crickets	flu	18/19	76	−30
S19	17	M	R	R>L	high tonal + high buzzing	Not known	NA	28	−12
S21	34	F	R	L>R	multiple high and low tones	Not known	9/4	76	−12
S22	47	M	L	L	hissing	started abruptly without reason	23/17	48	+2
S23	52	F	R	L = R	high tonal, hissing	Not known	NA	30	−2
S30	58	F	L	L = R	high tonal	Not known	58/116	66	+6
S37	59	M	R	L>R	high buzzing	sound exposure	14/7	92	+4
***Control***									
**ID**	**age**	**sex**	**handed-ness**	**T side**	**T type**	**T cause/initial event**	**PTA L/R**	**THI**	**ΔTHI**
CS6	63	F	R	L	siren, variable pitch	loss of balance, nausea	27/24	12	−2
CB9	68	F	R	L>R	hissing	Not known	28/21	16	+10
CB16	67	F	NA	NA	humming (~motor) + whistling	Not known	22/11	2	+6
CL40	29	F	L	R>L	whistling, ringing	Not known	−1/3	6	+2
CL41	64	M	R	L	high tonal	work-related sound exposure	43/22	10	+4

### TDCS device

**Fig 1:**
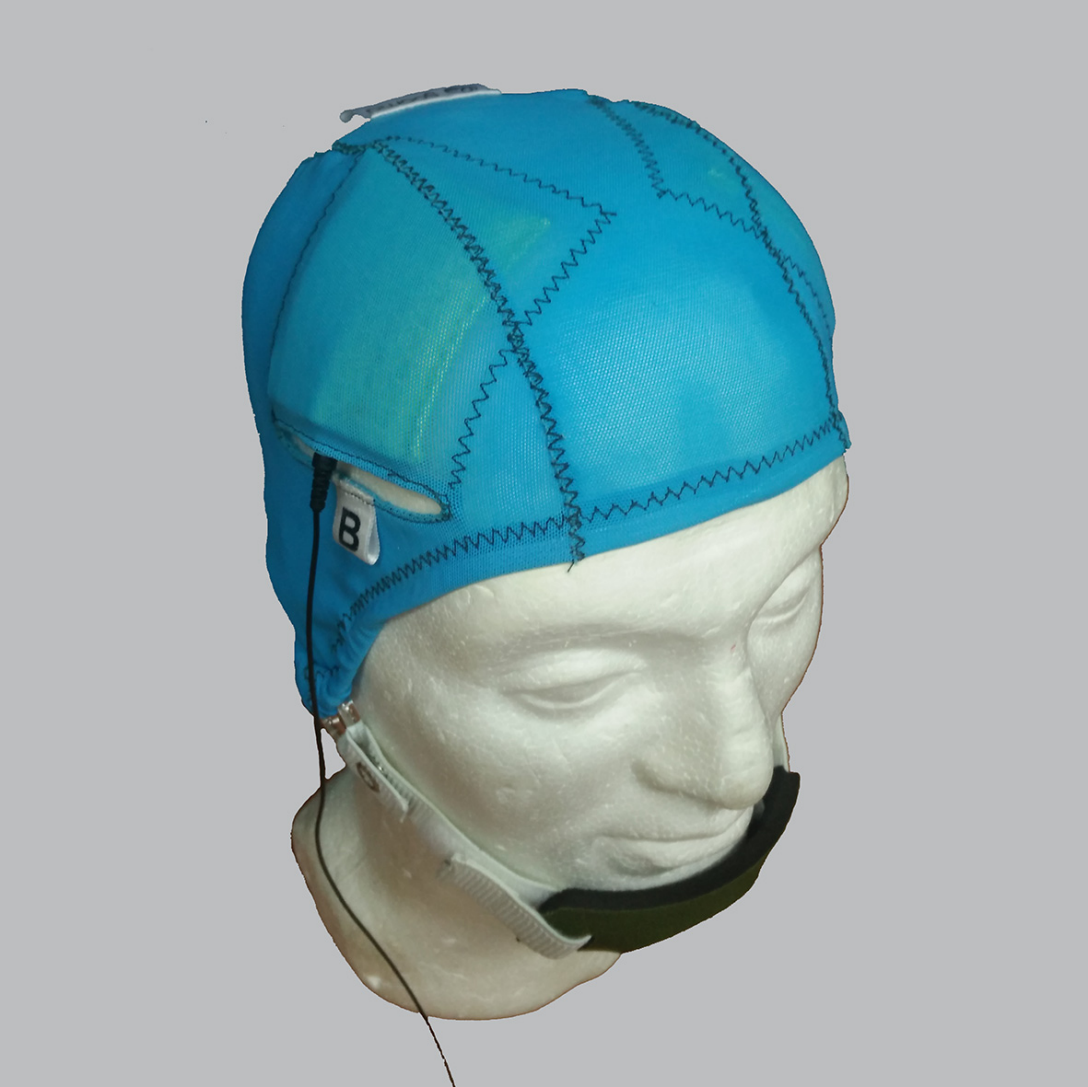
Custom-made tDCS electrode cap with sewn-on electrode positioning guides. Figure shows the cap for the bifrontal electrode montage. A different cap was used for the LTA montage.

A Sooma tDCS^TM^ device (Sooma Oy, Helsinki, Finland) was used in the study. The device is designed and approved for patient use with pre-programmed treatment parameters and hardware-level safety limits. Patients were given a package consisting of the stimulator unit and stimulation electrodes (consisting leads and pads) along with three pairs of sponge pouches for the electrode pads, a head cap with openings for the electrodes (Fig 1), a chinstrap and 0.9% saline solution.

### TDCS treatment

Treatment consisted of ten 20-minute tDCS sessions carried out on consecutive days. The first session was completed at the outpatient clinic after a training session, and the nine following sessions took place at home. The aim of the training session was to assure that patients were capable of carrying out the necessary preparations, which consisted of 1) soaking the electrode sponge pockets in saline and placing the stimulating electrodes inside the pockets; 2) while wearing a custom-made EEG cap where electrode locations had been marked, placing the electrodes under the cap correctly; 3) turning the device on; 4) using the device to check contact impedance of electrodes and, if necessary, adjusting the electrodes in order to keep impedance below 15 kΩ; 5) running the treatment; and 6) cleaning the electrode pockets and electrodes, making the device ready for the next treatment. All the stimulation parameters were pre-programmed into the tDCS stimulator so that when operating the device, the patient only had to press one button twice: first for starting the impedance check, and a second time for starting the treatment. Patients kept a treatment diary where they filled in the time of each session, along with free-form notes about any possible difficulties they might have had during the preparation or stimulation. They were also instructed to monitor the skin under the electrodes and to report back to the research staff in case there was long-lasting redness or visible damage in the skin.

TDCS was applied at a current of 2 mA, except in sham treatment (probability 33%) where the current was applied in the following way: 1) current was ramped up linearly from 0 mA to 2 mA during 20 seconds, and then 2) ramped down to 0.3 mA during 17 seconds, 3) kept at 0.3 mA (impedance measurement current) for the rest of the 20-minute treatment session, and finally 4) ramped down to 0 mA in 3 seconds. This procedure was used in order to produce a sensation identical to active treatment and to maintain constant impedance feedback. If the patient were to remove or adjust the electrodes or the cap mid-treatment, this protocol allowed the device to respond reliably, thus maintaining the blinding even in non-standard situations. Inter-electrode impedance was kept below 15 kΩ throughout the whole treatment session; in case the impedance exceeded the limit during the session, stimulation was interrupted and could only be resumed when impedance was brought within the accepted range by e.g. re-adjusting the electrodes.

Two different electrode placements were used in the study: left temporal area (LTA) and bifrontal montages. In LTA the 35 cm^2^ anode was placed over the left temporal area—targeting the auditory cortex—and the 50 cm^2^ cathode was placed contralaterally over the frontal area. In the bifrontal montage the anode and cathode were both 35 cm^2^ and placed symmetrically bilaterally over the frontal areas: anode on the left and cathode on the right side.

### Outcome measures

The main outcome measure of the study was the Tinnitus Handicap Inventory (THI) [26]. Secondary measures were the mini-Tinnitus Questionnaire (mTQ) [27], Beck Depression Inventory (BDI-IA) [28], Beck Anxiety Inventory (BAI) [29], and visual analog scales (VASs) for tinnitus loudness and annoyance. All measures were obtained during the first visit to the outpatient clinic, prior to the training and first treatment session, and again during a post-treatment visit four weeks (28 days) after the first session. The absolute values from the questionnaires were compared pre- to post-treatment with paired t-tests and the effect of treatment type was assessed by a one-way ANOVA, taking the pre- to post-treatment difference in outcome measure as the response variable. Previous studies have suggested that female patients as well as patients with less hearing loss might respond more favorably to tDCS treatment. Thus, an additional t-test was performed, comparing THI changes between male and female patients. Effect of hearing loss was assessed by calculating the Pearson’s product moment correlation coefficient between the PTA of the worse ear (the one with the higher PTA) and the THI change.

Patients also answered a non-standard questionnaire mapping the user experience aspects of the trial. There were eight yes/no-questions regarding the treatment: Q1) “Did the treatment have any effect on tinnitus loudness?”, Q2) “Did the treatment have any effect on tinnitus annoyance?”, Q3) “Did the treatment have any effect on your mood?”, Q4) “Did the treatment cause any adverse effects?”, Q5) “Did you experience any uncomfortable sensation on your skin during treatment?”, Q6) “Based on your user experience, was the device you were given a sham device?”, Q7) “Did the treatment have any effect on you falling asleep or on the quality of sleep?”, Q8) “Was it difficult to prepare the device and accessories before treatment?”. Patients were also encouraged to elaborate their answers in free-form. These answers were collected right after the ten-day treatment period had finished.

## Results

Out of the 43 patients, three did not complete the ten-day treatment period. Apart from these three patients, all other patients completed all ten treatment sessions and had marked them accordingly in their treatment diaries. Reasons for discontinuing the treatment were: perceived increase in tinnitus loudness in two cases (B15, B17) and a slight skin burn caused by incorrect use of electrodes in one case (L35). Five more patients had to be excluded from final analysis: three patients failed to provide data for the four-week post-treatment time point (L4, L27, L35), one patient had used the device incorrectly (B43, device was idling and not active during some of the treatment sessions), and one patient had started new medication for depression during treatment so that the effects of medication and tDCS could not be differentiated (L39). In total, out of the 43 patients who started the treatment, data from 35 patients were included in analysis.

After decoding, the following distribution of patients to different treatment groups was found: ten patients received active LTA stimulation (group *LTA*), nine patients active bifrontal stimulation (group *bifrontal*), and 11 patients sham stimulation using either LTA or bifrontal montage (group *sham*). Five patients were assigned to the *control* group (THI less than 18): two of them had active LTA, two had active bifrontal, and one had sham treatment with the LTA montage.

### Effect of tDCS on tinnitus

**Fig 2:**
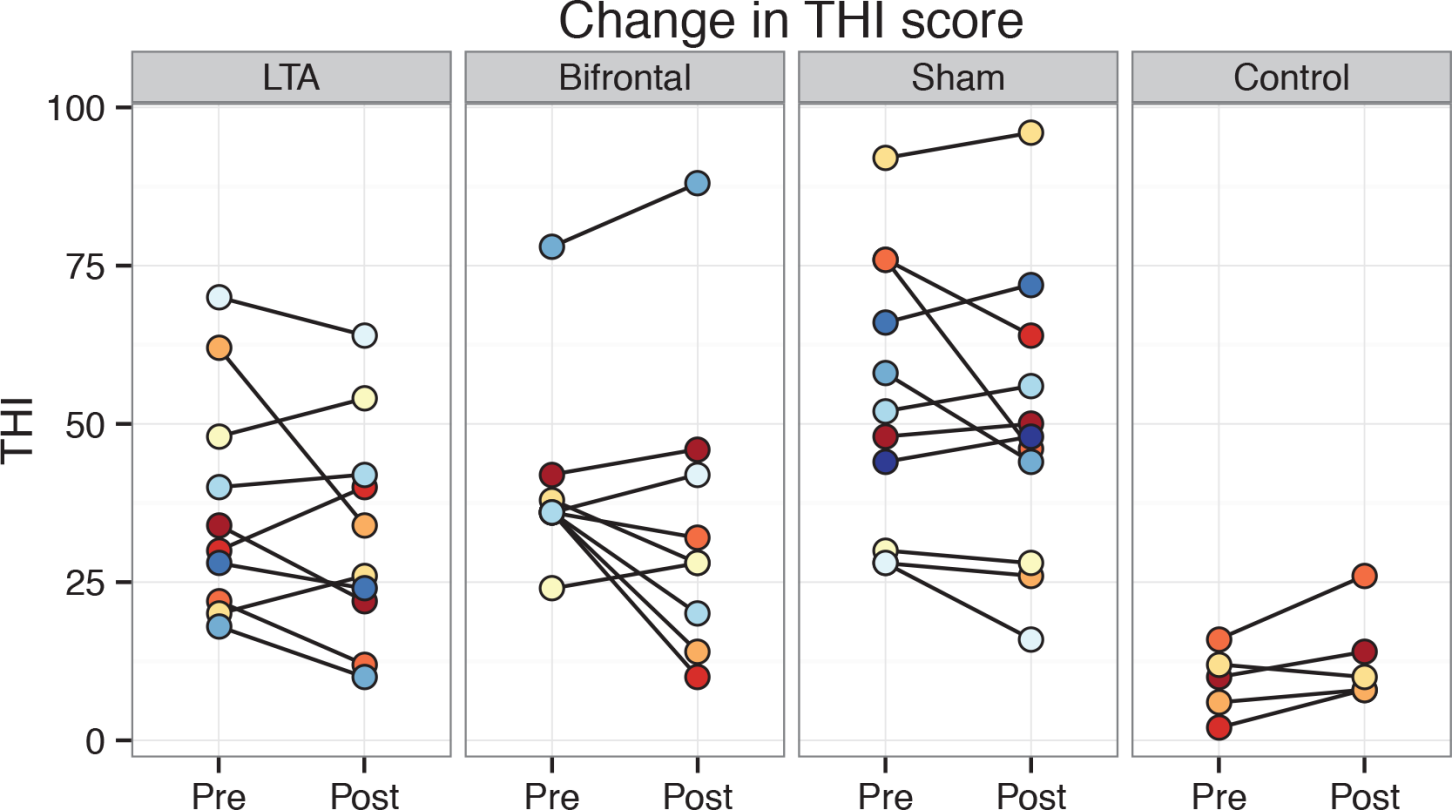
Individual THI scores pre- and post-treatment shown group-wise.

Baseline values for the THI ranged from 18 to 92 in the non-control groups and did not differ significantly between groups (Fig 2). Overall, THI scores in the non-control groups decreased significantly from pre- to post-treatment (mean change *−*5.0, *t(29) = −2*.*41*, *p < 0*.*05*). However, there was no effect of tDCS variant on the THI change (Table 2). The sample sizes needed to achieve statistical significance would be calculated in thousands (Cohen’s *d* for *LTA* vs *sham*: 0.03; and for *bifrontal* vs *sham*: 0.1). In the *control* group THI scores increased in all subjects except for the one who had received sham treatment. Male and female patients in *LTA* and *bifrontal* groups did not differ significantly in their THI changes (females: *−3*.*2*, males: *−7*.*3*, *t(17*.*0) = 0*.*76*, *p = 0*.*5*), nor did the degree of hearing loss have any significant correlation with the THI change (*r = −0*.*21*, *t(16) = −0*.*86*, *p = 0*.*4*).

**Table 2:** Group-wise average (± standard deviation) tinnitus scores.

	THI			mTQ			Loudness VAS			Annoyance VAS		
	*pre*	*post*	*Δ*	*pre*	*post*	*Δ*	*pre*	*post*	*Δ*	*pre*	*post*	*Δ*
*LTA*	37.2 ±17.8	32.8 ±17.5	*−*4.4 ±11.2	8.6 ±3.6	8.2 ±3.6	*−*0.4 ±2.8	5.3 ±2.0	4.8 ±1.8	*−*0.5 ±1.7	4.3 ±2.3	3.7 ±1.7	*−*0.5 ±1.8
*Bifrontal*	40.2 ±15.0	34.2 ±23.4	*−*6.0 ±13.1	10.9 ±4.2	9.9 ±4.1	*−*1.0 ±3.0	6.2 ±2.4	6.2 ±2.2	0.0 ±1.9	7.0 ±2.2	5.8 ±2.5	*−*1.2 ±3.4
*Sham*	54.4 ±21.5	49.6 ±22.6	*−*4.7 ±11.1	13.8 ±5.2	12.5 ±5.5	*−*1.4 ±2.7	6.4 ±2.3	6.2 ±2.3	*−*0.2 ±0.9	6.7 ±2.8	6.2 ±2.7	*−*0.5 ±1.0
*F(2*,*27)*	2.57		0.05	3.67		0.3	0.68		0.3	3.67		0.3
*p-value*	.1		.95	.04*		.7	.5		.7	.04*		.8
*Control*	9.2 ±5.4	13.2 ±7.6	+4.0 ±4.5	5.0 ±2.1	5.0 ±3.0	0.0 ±3.1	3.3 ±2.7	3.9 ±3.6	+0.7 ±1.7	0.6 ±0.8	1.2 ±1.5	+0.6 ±0.9

The Δ-column shows the average intra-individual change from pre- to post-treatment. The F-test compares scores between the LTA, bifrontal and sham groups.

* statistically significant at 5% significance level

**Fig 3:**
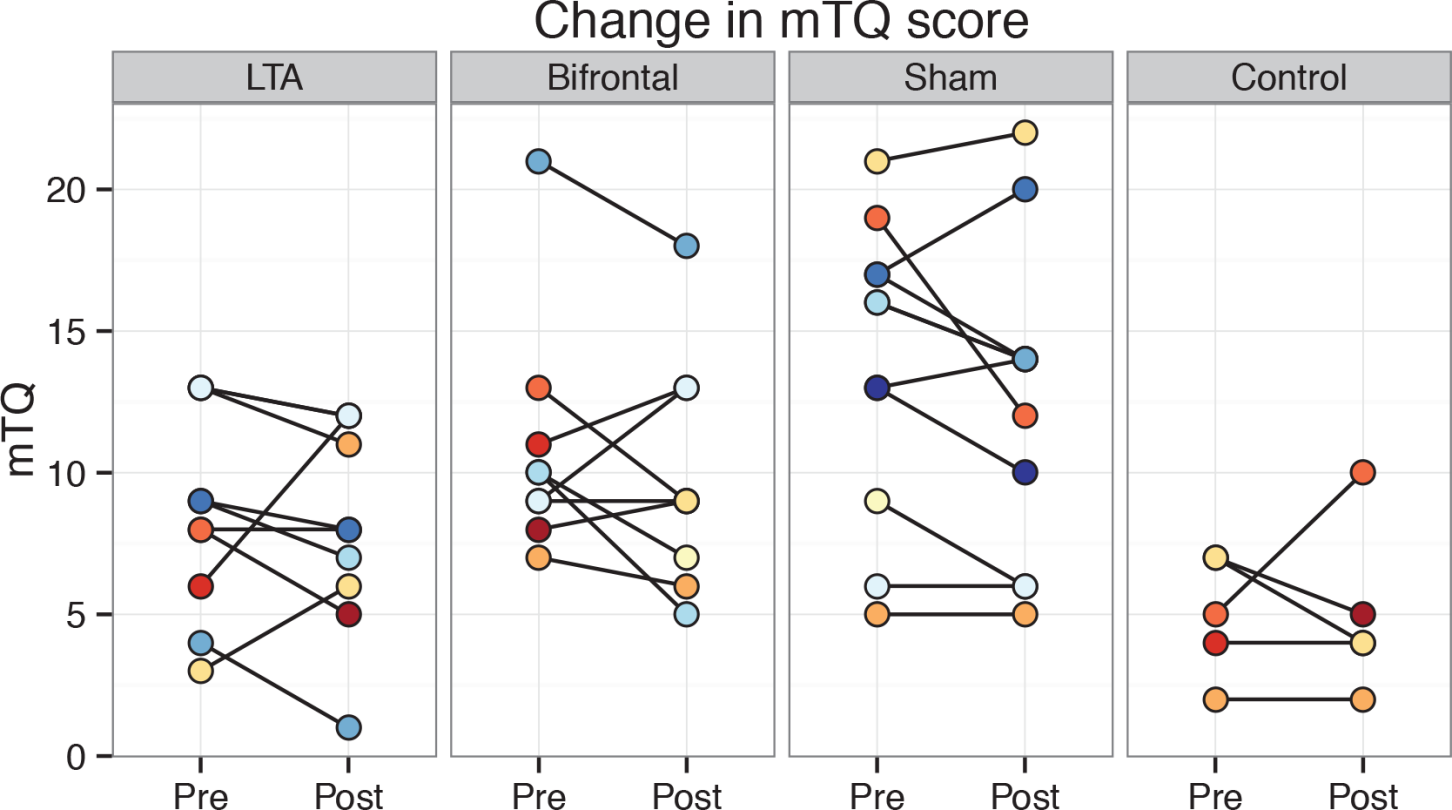
Individual mTQ scores pre- and post-treatment shown group-wise.

**Fig 4:**
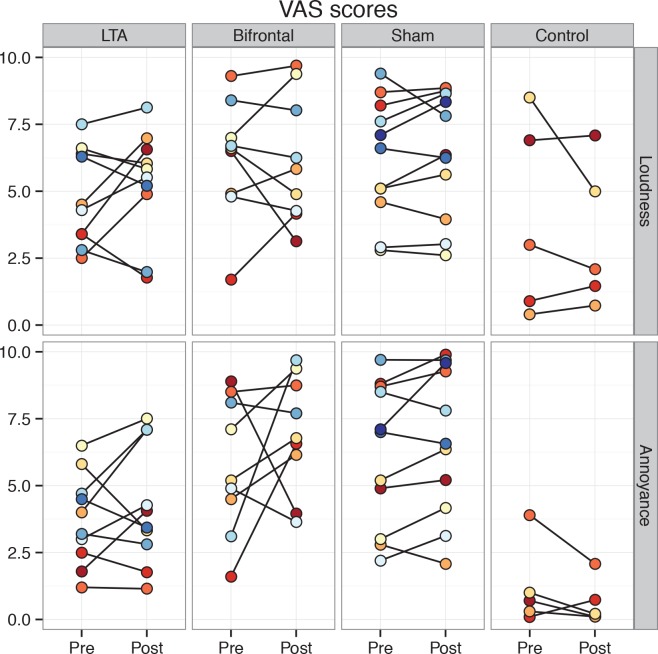
Individual VAS scores pre- and post-treatment shown group-wise.

Results for the secondary tinnitus outcome measures followed the same pattern as the THI, showing no significant differences between tDCS variants (Fig 3 & Fig 4). For the pre-treatment mTQ and annoyance VAS scores, there were significant differences between non-control groups. Pairwise post-hoc testing between the three groups revealed a significant difference between *LTA* and *sham* groups in mTQ. For the annoyance VAS, pairwise tests approached significance when comparing *LTA* to the other two groups (*LTA* vs. *bifrontal*: *p = 0*.*06*, *LTA* vs. *sham*: *p = 0*.*07*).

### Effect of tDCS on depression and anxiety

Depression and anxiety scores were relatively low in all groups, with mean scores for both BDI and BAI well below 10 out of the maximum of 63. Also the changes in BDI and BAI were minimal and showed no differences between non-control groups (Table 3). *Control* group had smaller BDI and BAI scores than the non-control groups, but the average changes in scores—an increase in BDI and a decrease in BAI—were larger than in any of the other groups.

**Table 3:** Group-wise average (± standard deviation) depression (BDI) and anxiety (BAI) scores. The Δ-column shows the average intra-individual change from pre- to post-treatment. The F-test compares scores between the LTA, bifrontal and sham groups.

	BDI			BAI		
	*pre*	*post*	*Δ*	*pre*	*post*	*Δ*
*LTA*	6.6 ±3.7	6.3 ±4.3	*−*0.3 ±2.0	4.2 ±3.4	3.4 ±3.6	*−*0.8 ±2.1
*Bifrontal*	8.9 ±10.4	8.8 ±12.2	+0.2 ±3.1	5.0 ±3.5	4.6 ±1.9	*−*0.4 ±2.5
*Sham*	8.4 ±9.0	8.3 ±9.9	*−*0.1 ±4.1	6.6 ±5.9	5.4 ±7.0	*−*1.3 ±3.4
*F(2*,*27)*	0.17		0.06	0.80		0.23
*p-value*	.8		.9	.5		.8
*Control*	2.0 ±1.2	3.4 ±5.0	+1.4 ±4.4	2.4 ±2.5	0.8 ±0.8	*−*1.6 ±2.2

### User experience

In general, patients found the treatment easy to administer and had relatively neutral experiences from the trial. Looking at the questionnaire answers, out of the 35 patients who completed the treatment and provided post-treatment data, only five patients (B11, L13, CS16, B26, and B29) reported that the treatment had a positive effect on tinnitus loudness (Q1, see *Outcome Measures* in section *Materials and Methods*) and six (B11, L13, CB16, S21, B26, and B29) felt that the treatment had a positive effect on tinnitus annoyance (Q2). The only patients reporting increases in tinnitus loudness and annoyance discontinued the treatment (B15, B17). One of them (B17) completed the post-treatment questionnaires. Tinnitus distress scores had decreased very remarkably for this patient: THI from 66 to 14, and mTQ from 11 to 4. All other patients reported no changes in tinnitus loudness or annoyance during the treatment. Four patients (B1, L20, S21, and B26) experienced that the treatment affected their mood in a positive way (Q3), whereas one patient (L42) felt more irritated than usual during the treatment. Five patients had had effects associated with the treatment that they considered mildly aversive (Q4): waking up in the night and not being able to fall asleep again for a while (CL40), overall increased sleepiness (B11), mood changes (L42), and the tingling sensation during stimulation (L13, L32). Twenty patients (B1, B7, L8, CB9, L10, B11, S12, L13, CB16, S19, S21, B25, B26, L28, L31, L32, B36, S37, CL40, L42) felt an uncomfortable sensation on the skin during stimulation (Q5), but all considered it to be well tolerable and to fade away after the first few minutes. Three patients experienced worse sleep during the treatment (Q7) evidenced by waking up in the night (S22, CL40), and nightmares (L42); and four patients felt that the treatment had a positive effect on their sleep (B7, B11, S21, and B29). Only six patients (B7, CB9, L10, S19, S21, and L32) felt that the treatment preparations were difficult to complete (Q8).

The successfulness of blinding to active vs. sham treatment was assessed by asking the patients which treatment they thought they got (Q6). Out of the 24 patients who received active stimulation, nine guessed correctly (B7, L8, B11, B24, B26, B36, L38, CL40, CL41), nine guessed they received sham treatment (B1, L2, CB9, L10, L13, CB16, L20, L32, B34), and six patients did not answer or gave an ambiguous answer (e.g. by choosing both ‘yes’ and ‘no’ options). In the *sham* group, seven patients out of 11 guessed correctly that they received non-active treatment (S3, S5, S12, S14, S18, S19, S23), two assumed that they had an active device (S21, S22), and two gave no answer. In other words, in the active treatment groups there were equally many correct (37.5%) and incorrect (37.5%) answers, whereas in the *sham* group there were remarkably more correct guesses (63.6%): it was twice as likely to guess the treatment variant correctly in the *sham* group than in the active treatment groups. However, the 2x3 Fisher’s exact test for count data did not indicate statistically significant results between the answer counts in active and *sham* groups (*p > 0*.*4*).

## Discussion

Effects of a ten-day at-home tDCS treatment on tinnitus as well as the safety and feasibility of the protocol were investigated in a double-blind sham-controlled setting. A small improvement in the main outcome measure, THI, was found, but the observed effect did not differ between active (2 mA) and sham (0.3 mA) treatment groups, suggesting that the decrease in THI scores was a non-specific sham effect, possibly related to the domiciliary treatment setting.

Self-administered tDCS treatment was safe and well tolerated among all patients. The only minor aversive event (slight skin burn) was due to an incorrect placing of the cathode electrode in the LTA montage. In this case it is likely that the wrong side of the electrode was, by mistake, facing the scalp in such a way that a small mound on the electrode surface—covering the stimulator wire terminal—could have caused an uneven distribution of current under the electrode. This uneven contact with the scalp would then have resulted in a higher current density under the wire terminal. The patient did not notice the error during the treatment session, but only on the following day. They then contacted the study personnel and were instructed to discontinue the treatment. This mild superficial burn required no medical attention and healed within a week, leaving no visible marks. The stimulator was sent to the manufacturer where it was inspected for any malfunctions but none were found. All devices were also reprogrammed by the manufacturer in order to keep the double-blinding effective. Although self-administered tDCS was found safe, it should be pointed out that proper training for the stimulation device is essential, and that pre-treatment screening is done by healthcare professionals in order to determine the subjective applicability of the treatment protocol. The authors want to stress that tDCS or other tES methods should not be used by individuals for self-medication purposes without recommendation and supervision by a healthcare professional.

The results regarding the changes in tinnitus distress are in line with recent double-blind sham-controlled tDCS treatment trials [30–32], where no advantage of active tDCS was found over sham treatment, using either LTA or bifrontal montages. In the present study there was a significant reduction in THI scores from pre- to post-treatment for non-control groups, contrary to the earlier sham-controlled trials and another open-label study by Frank et al. [18]. This effect did not differ between treatment variants, so it cannot be attributed to the tDCS treatment. Since such a decrease has not been observed in in-hospital treatment trials [30–32], one possible explanation for the overall improvement in tinnitus scores could be that the ability to use the device at home—instead of frequently visiting the clinic—may have had a positive impact on the general outcome of the study. No evidence could be found for a more beneficial treatment response in female patients, contrary to the results of Frank et al. [18]. In fact, in the current study male participants reported a greater average reduction in THI scores than females. Also, the results of Fregni et al. [19] could not be confirmed, since no correlation was found between the treatment response and hearing ability—as measured by the PTA of the worse ear.

It was assumed that the weak 0.3 mA impedance measurement current used during the sham protocol would have no treatment effect. The 0.3 mA measurement current was applied in order to achieve the most realistic sham condition from the user’s perspective, providing immediate electrode contact feedback. The impedance measurement current intensity was experimentally determined so that the resulting impedance estimate was stable and reliable throughout the sham treatment session. Weaker currents were found to give less coherent impedance estimates. Since the at-home treatment sessions were not supervised, the device had to perform identically in both active and sham conditions. In theory it could be possible that even small current intensities would yield a therapeutic effect. Weak currents (< 0.5 mA) have shown potential for modulating brain excitability [33–35]. Weak tDCS currents have also been used as a sham condition in a single-session study [36], but to the authors’ knowledge there are no previous treatment studies involving a weak current control condition. When comparing 1 mA and 2 mA LTA tDCS, Shekhawat, Stinear & Searchfield (2013) [37] found a significantly greater immediate suppression of tinnitus loudness for 2 mA stimulation, with weak to non-existent effects for 1 mA stimulation. Thus, even if the 0.3 mA sham stimulation would result in a treatment effect, it would most likely be smaller compared to the 2 mA treatment. Since recent tDCS studies have found no effect even for treatment with 2 mA stimulation [30–32], it is unlikely that the sham condition used in the current study would result in a therapeutic effect. However, as in the current study there was an overall decrease in THI scores when the active and sham groups are pooled, the possibility of an effect for 0.3 mA treatment effect cannot be completely ruled out.

For the control group, THI scores increased on average, although four patients felt that the treatment did not have any effect on their tinnitus and one reported improvements in both tinnitus loudness and annoyance in the user experience questionnaire. However, these improvements were not reflected in the THI or mTQ scores. Naturally, at the lower end of the scale there are constraints as to how much the THI score can decrease, even in the case when tinnitus would be completely abolished. Thus, THI may not be the optimal measure for quantifying treatment outcome in these patients. Since THI is, however, widely used and preferred as an outcome measure in tinnitus trials [38], it may be beneficial to exclude subjects with low THI scores from treatment trials.

The changes in THI scores can also be viewed from another perspective, namely by looking at the number of patients whose tinnitus improved or worsened by a clinically significant amount as a result of the treatment. A change of more than seven points on the THI scale has been found to yield a clinically significant change in tinnitus distress [39]. In the *LTA* group there were four clinically significant improvements and one significant worsening in THI scores; four improvements and one worsening in the *bifrontal* group; four improvements in the *sham* group; and one worsening in the *control* group. Also from this perspective, all the groups show more positive changes than negative, but again the effect cannot be linked to active tDCS.

Secondary outcome measures followed the same pattern as the main outcome measure THI, showing no clear evidence for differential effects between tDCS variants. Looking at the VAS scores, one might be able to find limited support for the hypothesis that LTA affects more the loudness of tinnitus, whereas bifrontal stimulation might modulate the affective dimensions of tinnitus. In the current study, LTA stimulation affected both loudness and annoyance of tinnitus, but bifrontal stimulation had no effect on loudness and a larger effect on annoyance. Depression and anxiety scores were relatively low in all patients, and no dramatic changes were seen in any of the groups. The majority of patients had BDI scores corresponding with minimal depression symptoms (25 patients with less than 10 points) [40] and all patients had BAI scores under 21 points, indicating a low grade of anxiety.

The majority of user feedback was either neutral or positive. Out of the 35 patients who completed the treatment and provided post-treatment data, only six felt that the stimulation was difficult to apply. An interesting and important—although not statistically significant—result from the user experience questionnaires is the successfulness of blinding to active or sham stimulation. In the *sham* group it was roughly twice as likely to guess the tDCS variant correctly than in the active treatment groups. Although most of the patients seemed to base their guess on the perceived success of the treatment (i.e. if the patient did not feel that the treatment had a positive effect, they would guess that they received sham stimulation in the hope that active stimulation would yield a better result), many patients in the active treatment groups also referred to maintained tingling sensations during the stimulation. Since all patients were naïve to tDCS, their answers could not have been based on earlier experience with the treatment. Further, in the active treatment groups, patients’ answers divided equally into correct and incorrect guesses, whereas in the *sham* group correct answers were more frequent. This suggests that there could be room for improvement in the sham stimulation protocol, possibly taking into account lasting skin sensations. The approach introduced by Pal et al. [32] takes this into account by using a small electrode pair in a single electrode site, preventing the current flow from reaching the brain while providing tactile stimulation identical to the active stimulation. However, some adjustments to the electrodes and the stimulator device would have to be made in order to apply this approach in a domiciliary setting.

The current study is limited in that the immediate effects of tDCS were not assessed. Thus, comparison with earlier studies on effects of single-session tDCS on tinnitus cannot be done. Future studies should include indicators for immediate effects, for example by comparing loudness or annoyance VASs before and after each treatment session. Another clear limitation of the current study is the small sample size, which limits the analysis regarding differences between tDCS variants. Although even remarkable increases in sample sizes might not provide significant results on a group level, larger groups could reveal subgroups of patients who benefit from treatment perhaps more than others. The patient groups were also relatively heterogeneous, with different types of tinnitus and varying hearing loss profiles present in all groups. Thus, the negative findings of the current study could probably be at least partly explained by interindividual variability in treatment response. Finding the most promising treatments for different tinnitus subtypes still poses a major challenge in tinnitus research. Increased flexibility in treatment trial participation—such as the possibility to use the treatment equipment autonomously at home—could aid in identifying the most relevant patient groups for different tinnitus treatments. Finally, it must be noted that the stimulation device did not have logging functionalities which would have allowed the research personnel to verify the patients’ compliance to the treatment. Future domiciliary studies could benefit from a device that would automatically log the essential treatment parameters, such as time of day, electrode impedance, and possible inconsistent events during a session.

## Conclusions

Self-administered at-home tDCS was safe and easy to use and gave similar results in tinnitus outcome measures to recent in-hospital trials. When proper training is given to the patients, self-administered tDCS with a pre-configured device is a feasible way for conducting long-term tDCS treatment trials, and more importantly, this is not restricted to tinnitus only. There was no beneficial effect of active (2.0 mA) tDCS treatment on tinnitus distress when compared to sham (0.3 mA) treatment.
